# Pharmacological modulation of Kv1.3 potassium channel selectively triggers pathological B lymphocyte apoptosis in vivo in a genetic CLL model

**DOI:** 10.1186/s13046-022-02249-w

**Published:** 2022-02-16

**Authors:** Filippo Severin, Andrea Urbani, Tatiana Varanita, Magdalena Bachmann, Michele Azzolini, Veronica Martini, Marco Pizzi, Angelo Paolo Dei Tos, Federica Frezzato, Andrea Mattarei, Paolo Ghia, Maria Teresa Sabrina Bertilaccio, Erich Gulbins, Cristina Paradisi, Mario Zoratti, Gianpietro Carlo Semenzato, Luigi Leanza, Livio Trentin, Ildiko Szabò

**Affiliations:** 1 Department of Medicine, Hematology and Clinical Immunology Branch, University of Padua School of Medicine, Padua, Italy and Veneto Institute of Molecular Medicine (VIMM), Padua, Italy; 2 Department of Biomedical Sciences, University of Padua, Padua, Italy; 3 Department of Biology, University of Padua, Padua, Italy; 4 Department of Medicine, Pathology Branch, University of Padua School of Medicine, Padua, Italy; 5 Department of Pharmaceutical and Pharmacological Sciences, University of Padua, Padua, Italy; 6 Università Vita-Salute San Raffaele and IRCC Ospedale San Raffaele, Milan, Italy; 7 Department of Experimental Therapeutics, The University of Texas MD Anderson Cancer Center, Houston, TX USA; 8 Department of Molecular Biology, University of Duisburg-Essen, Essen, Germany; 9 Department of Chemical Sciences, University of Padua, Padua, Italy; 10 CNR Institute of Neurosciences, University of Padua, Padua, Italy

**Keywords:** Ion channels, Chronic lymphocytic leukemia, Drug resistance, Mitochondria, Apoptosis

## Abstract

**Background:**

Ion channels are emerging as promising oncological targets. The potassium channels Kv1.3 and IKCa are highly expressed in the plasma membrane and mitochondria of human chronic lymphocytic leukemia (CLL) cells, compared to healthy lymphocytes. In vitro, inhibition of mitoKv1.3 by PAPTP was shown to kill ex vivo primary human CLL cells, while targeting IKCa with TRAM-34 decreased CLL cell proliferation.

**Methods:**

Here we evaluated the effect of the above drugs in CLL cells from ibrutinib-resistant patients and in combination with Venetoclax, two drugs used in the clinical practice. The effects of the drugs were tested also in the Eμ-*TCL1* genetic CLL murine model, characterized by a lympho-proliferative disease reminiscent of aggressive human CLL. Eμ-*TCL1* mice showing overt disease state were treated with intraperitoneal injections of non-toxic 5 nmol/g PAPTP or 10 nmol/g TRAM-34 once a day and the number and percentage of pathological B cells (CD19^+^CD5^+^) in different, pathologically relevant body districts were determined.

**Results:**

We show that Kv1.3 expression correlates with sensitivity of the human and mouse neoplastic cells to PAPTP. Primary CLL cells from ibrutinib-resistant patients could be killed with PAPTP and this drug enhanced the effect of Venetoclax, by acting on mitoKv1.3 of the inner mitochondrial membrane and triggering rapid mitochondrial changes and cytochrome c release. In vivo, after 2 week- therapy of Eμ-*TCL1* mice harboring distinct CLL clones, leukemia burden was reduced by more than 85%: the number and percentage of CLL B cells fall in the spleen and peritoneal cavity and in the peripheral blood, without signs of toxicity. Notably, CLL infiltration into liver and spleen and splenomegaly were also drastically reduced upon PAPTP treatment. In contrast, TRAM-34 did not exert any beneficial effect when administered in vivo to Eμ-*TCL1* mice at non-toxic concentration.

**Conclusion:**

Altogether, by comparing vehicle versus compound effect in different Eμ-*TCL1* animals bearing unique clones similarly to CLL patients, we conclude that PAPTP significantly reduced leukemia burden in CLL-relevant districts, even in animals with advanced stage of the disease. Our results thus identify PAPTP as a very promising drug for CLL treatment, even for the chemoresistant forms of the disease.

**Supplementary Information:**

The online version contains supplementary material available at 10.1186/s13046-022-02249-w.

## Background

B cell tumors represent a major class of lymphoproliferative disorders. In particular, the B-cell chronic lymphocytic leukemia (CLL) has high incidence among adults in the Western countries, being the most frequent type of leukemia. This clinically heterogeneous disease is characterized by a relatively homogeneous transcriptional profile and immunophenotype, and by an aberrant accumulation of mature, apoptosis-resistant CD19^+^CD5^+^ monoclonal B-lymphocytes in peripheral blood [1]. To date, there are different novel CLL-targeting drugs that improved the clinical outcome and act on specific kinases or on the apoptotic mechanism (e.g. [2]). Ibrutinib inhibits Bruton’s tyrosine kinase (BTK), a key player in B-cell receptor (BCR) signaling that regulates B-cell growth. This drug is effective also against aggressive forms of CLL with mutations of the tumor suppressor *TP53* [3], whose activation is required to trigger the intrinsic apoptotic pathway. By inhibiting BTK, ibrutinib prevents re-expression of sufficient levels of functional surface membrane chemokine receptors (e.g. CXCR4) and thereby causes failure of CLL B cells to re-enter niches, leading to greater cell death and delay in CLL disease progression [4, 5]. Ibrutinib was also shown to exert a pro-apoptotic effect (see e.g. [6, 7]). Venetoclax instead acts by chemically inhibiting the mitochondrial anti-apoptotic Bcl-2 protein, whose expression is upregulated in the majority of the patients [8]. Unfortunately, not all patients treated with these drugs respond or remain disease-free and some of them relapse or become resistant [8–10]. Therefore, novel therapeutic strategies and approaches for drug discovery and development are nowadays unmet clinical needs.

Today, ion channels are recognized as important contributors to tumor development and progression on the basis of both in vitro and in vivo data (e.g. [11–13]), thus they are considered important oncological targets as well as possible cancer biomarkers [14]. Among the several types of ion channels, calcium and potassium ones play a pivotal role as important regulators of cell cycle and proliferation [12, 15–17]. Both myeloid and lymphoid leukemic cells express high levels of several potassium channels, including the voltage-gated potassium channel Kv1.3 (encoded by *KCNA3*). This channel is expressed in the plasma membrane (PM) of healthy lymphocytes, and to a higher extent in the PM of pathological ones [18–21]. The calcium-activated intermediate-conductance potassium channel IKCa, also called KCa3.1 (encoded by *KCNN4*), is also highly expressed in B cells isolated from patients with CLL [20, 22]. IKCa opening enhances the driving force promoting the entry of calcium into these cells, with consequent triggering of proliferation [23]. A small-molecule membrane-permeant inhibitor of IKCa, triarylmethane-34 (TRAM-34) [24], has recently been shown to decrease proliferation of activated ex vivo human CLL B cells [20].

Beside PM-located channels and transporters, intracellular, especially mitochondrial ion channels have been identified as promising oncological targets as well [25, 26]. Interestingly, and similarly to IKCa, Kv1.3 was found to be active also in the mitochondrial inner membrane (mtKv1.3) in several cell types [27], including leukemic T lymphocytes [28] and ex vivo primary leukemic B cells isolated from patients [21]. The mitochondria-targeted selective mtKv1.3 inhibitor PAPTP was able to trigger apoptosis and specifically eliminate in vitro primary CLL B cells isolated from patients [29]. PAPTP, a chemical derivative of the selective Kv1.3 small molecule inhibitor PAP-1 [30], harbors a positively-charged triphenylphosphonium moiety that allows accumulation of the drug into mitochondria [29]. Since the primary sequences of human and murine Kv1.3 are highly similar, PAPTP efficiently exerts its effects in both human and murine cell lines expressing Kv1.3 [29, 31]. Specificity of its action is indicated by lack of changes in mitochondrial parameters and death upon silencing of Kv1.3 in murine melanoma cells [29]. In contrast, inhibitors that target the PM-located Kv1.3, namely Charybdotoxin and Stichodactyla toxin ShK, did not have any effect on cell death [29, 32].

Starting from these in vitro observations, we aimed at testing the two channel-targeting drugs PAPTP and TRAM-34 in vivo in the Eμ-*TCL1* mice, where the overexpression of *TCL1* driven by the IgM heavy chain enhancer leads to the development of a B-cell leukemia closely resembling the human, aggressive, treatment-resistant CLL. These animals develop a monoclonal CD5^+^CD19^+^ B-cell lymphocytosis with biologic and clinical features similar to human CLL [33–35]. Importantly, gene rearrangement analysis proved that the same CD5^+^ B cell clone localizes to bone marrow, spleen and peripheral blood in Eμ-*TCL1* mice [36]; this observation underlines that a direct comparison of the effects of a drug on the same leukemic cells in different tissue compartments of these animal can be carried out. Indeed, Eμ-*TCL1* mouse is a well-characterized model (e.g. [33, 34, 37] that has been widely used by the scientific community to assess the effects of drugs with potential or known therapeutic effects. Drugs can be tested either directly in Eμ-*TCL1* mice or using the adoptive transfer model, where a single clone from an Eμ-*TCL1* mice is transferred to a number of WT animals in order to collect data on drug response from a high number of animals and to reduce variability (e.g. [3, 34, 38–41]). In our experiments, we opted for using individual Eμ-*TCL1* mice with overt pathological state to assess the effects of the used drugs, since this represents a situation that is more closely resembling the one encountered in the clinic: i) each individual Eμ-*TCL1* mice harbors a specific B-CLL clone, exactly as the patients do; ii) pathologic CLL cells develop and are sustained depending on signals from the tumor microenvironment (see e.g. [42, 43]). Our *in vivo* study suggests that mitochondrial channel-targeting drugs might be proposed as an additional choice for treatment of CLL.

## Materials and methods

### Patients, cell separation, and culture conditions

Peripheral blood B lymphocytes were derived from patients diagnosed with treatment-naïve CLL (if not specified otherwise) followed at Padua University Hospital and diagnosed according to international workshop of CLL 2018 guidelines [44]. Peripheral blood mononuclear cells of the patients were isolated by density-gradient centrifugation over Ficoll-Paque (GE Healthcare; Uppsala, Sweden). Where necessary, further purifications employed the RosetteSep isolation kit for B-cells (STEMCELL Technologies; Vancouver, Canada). The obtained purity of peripheral blood cells was at least 95% (CD19^+^CD5^+^), and was assessed by flow cytometry [45]. Purified cells (4 × 10^6^ cells/mL) were cultured in suspension in RPMI-1640 medium (EuroClone; Milan, Italy) supplemented with 2% heated inactivated Fetal Calf Serum (FCS; Invitrogen, Paisley, UK), 2 mmol/L-glutamine, 100 μg/ml penicillin and 100 μg/mL streptomycin in 24 or 96-well plates, at 37 °C in a humidified atmosphere containing 5% CO_2_ [46]. Cells were treated for 24 h with the described compounds at the indicated concentration.

### Cell counting

Cells' absolute number was determined by the use of Neubauer’s chamber (Optik Labor, Friedrichsdorf, Germany) and Türk’s solution (Merck, Darmstadt, Germany). Briefly, blood or cell samples were diluted 1:20 in Türk’s solution, then the counting chamber was filled with the suspension and cells were counted under the microscope with a × 10 objective.

### Cell viability evaluation

After culture, human or mouse cell apoptosis was assessed using one of these 2 methods: (i) staining with Annexin V-FITC kit (Immunostep; Salamanca, Spain), as previously described [46] and analysis with FACSCanto™ II cytometer (Becton Dickinson, Mountain View, CA, USA) or (ii) staining for 30 min at 37 °C with 1 μl of Annexin V mix (Merck, Darmstadt, Germany), resuspension in 500 μl HBSS and analysis using BD LSRFortessa™ X-20 Cell Analyzer.

### Western blotting analysis

500 × 10^6^ CLL B cells were washed in PBS, incubated for 30 min in mitochondrial isolation buffer (200 mM sucrose, 10 mM Tris-HCl, 1 mM EDTA) and lysed with a glass Potter-Elvehjem homogeniser. The sample was centrifuged at 1000 g for 10 min. After centrifugation of the supernatant (whole cell lysate) at 6500 g for 10 min, the pellet (containing the membrane-enriched fraction) was purified on a Percoll gradient to obtain mitochondria-enriched fractions. Twenty five microgram of whole cell lysates and mitochondria-enriched fractions were loaded on a precast gel (GenScript ExpressPlus™ PAGE Gel, 4–20%) and subjected to Western Blot. The following antibodies were used for protein detection: plasma membrane marker PMCA (Thermo Fisher Scientific, #MA3–914), mitochondrial marker TOM-20 (Santa Cruz Biotechnologies, #sc-11,415), KCa3.1 (Abcam, #ab229593).

### Mice and in vivo procedures

Eμ-*TCL1* mice were a kind gift from Prof. Croce (Columbia, New York) and were provided by the laboratory of Prof. Ghia (IRCCS Ospedale San Raffaele, Milan). Mice were maintained in a SPF animal house. Starting from 7 to 8 months old animals, blood samples were collected from the tail vein to determine the percentage of total lymphocytes and of CD19^+^CD5^+^ CLL cells. Mice underwent treatment when the percentage of CD19^+^CD5^+^ cells on the total lymphocytes was higher than 40%. Both PAPTP and TRAM-34 were solubilized in DMSO and injected intraperitoneally. Animals received a daily dose of the drugs (see text for quantity) or an equal amount of DMSO (on average 2% in PBS) for 5 days every week, for 2 weeks; generally, few microliters of the drug solution (in pure DMSO) were dissolved in saline solution and injected, leading to a percentage of DMSO on the animal weight of 0.12–0.16%/gbw, which is not toxic to the cells. Experiments on mice and human CLL B cells were conducted according to the Local Ethical Committee at the University of Padua and National Agency, and with the supervision of the Central Veterinary Service of the University of Padova (in compliance with Italian Law DL 116/92 and further modifications, embodying UE directive 86/609), authorization n. 4218/A0/17 and 111/2017-PR.

### Ex vivo experiments and flow cytometry

At the end of the experimental protocol (after 2 weeks of treatment), mice were sacrificed and blood, spleen, bone marrow and peritoneal cells were collected. Spleen volume was immediately assessed using a water-filled cylinder (hydrostatic weighing). Bone marrow cells were isolated by flushing the cavities of the femur and tibia with ice cold RPMI 1640 containing Penicillin-Streptomycin (Euroclone, Milan, Italy) and filtered with a cell strainer cap from Round-Bottom Tube (Corning Incorporated, Corning, NY, USA) (Invitrogen; Paisley, UK). Spleens were dissociated in RMPI 1640 medium utilizing a 40 μm Cell Strainer (Corning Incorporated). Peritoneal wash was performed post-mortem by flushing the abdominal cavity with RPMI 1640.

Cells were pelleted and re-suspended in PBS, counted in a Burker chamber and stained with the antibodies for FACS analysis. Normal B-, pathological B- and T- cells from Eμ-*TCL1* mouse tissues were identified by Flow Cytometry. 1 × 10^6^ cells were lysed with BD Pharm Lyse 10x (BD Biosciences, Heidelberg, Germany) lysing buffer for 5 min at room temperature and then were stained with the following antibodies for 30 min at 4 °C: anti-CD19 PE, anti-CD5 APC, anti-CD45 APC-Cy7 (1 μg of antibody in 100 μL of samples; BD Biosciences) and with Mouse T Lymphocyte Subset Antibody Cocktail (a solution with anti-CD3 PE-Cy7, anti-CD4 PE and anti-CD8 FITC, 10 μL of antibody in 100 μL of samples; BD Biosciences). Mitochondrial membrane potential and ROS production were measured with TMRM and mitoSOX, respectively, as previously described [29]. Cytochrome c release was assessed as described in [28].

### Kv1.3 expression

Peripheral blood samples from Eμ-*TCL1* mice were collected from the tail vein and lysed with BD Pharm Lyse 10x (BD Biosciences, Heidelberg, Germany) lysing buffer; peripheral blood samples from CLL patients were processed in the same way. Cells were stained as aforementioned, with anti-CD19 PE, anti-CD5 APC, anti-CD45 APC-Cy7 antibodies and the expression of Kv1.3 channel in lymphocytes was evaluated by flow cytometry using a FITC-labelled anti-Kv1.3 antibody against mouse or human Kv1.3 epitopes (Merck, Darmstadt, Germany). Ten microlitres of sample were incubated with 1 μg of antibody for 10 min at room temperature. Then samples were centrifuged and the supernatant was discarded. Before acquisition, cells were centrifuged, resuspended in 200 μL of PBS and scanned by a FACSCanto™ II cytometer (Becton Dickinson, Mountain View, CA, USA). Fluorescence Median Intensity (MFI) was considered. The data were processed using DIVA Software (Becton Dickinson).

### Hematoxylin-eosin staining

Tissue samples, obtained as described above, were processed for histology and stained with hematoxylin and eosin as previously reported [29]. Briefly, liver and spleen were removed, washed with RPMI and fixed in 4% paraformaldehyde in PBS for 36 h. Tissues were serially dehydrated, cleared with xylene and embedded in paraffin for sectioning at a thickness of 7 μm. Sections were then dewaxed, rehydrated and incubated for 4 min in 0.1 M citrate buffer (pH 6.0) in a microwave oven operating at 350 W. Sections were stained for 2 min with hematoxylin-eosin and washed with water prior to being mounted in Mowiol (Merck, Darmstadt, Germany). Images were acquired using a Leica TCS-SP2 microscope (Leica Microsystem, Wetzlar, Germany).

## Results

### CD19^+^/CD5^+^ leukemic B cells express high levels of Kv1.3 and are sensitive to PAPTP-triggered apoptosis

**Fig. 1:**
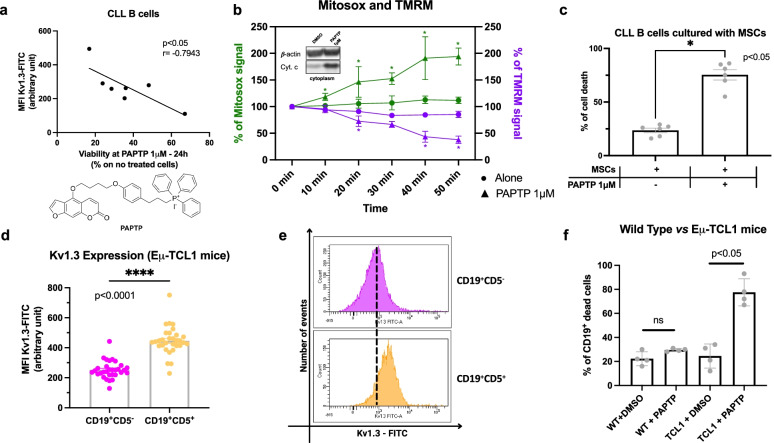
Kv1.3 expression and effect of PAPTP on pathological human and murine lymphocytes ex vivo. **a** Kv1.3 expression was assessed by flow cytometry on ex vivo CLL B cells isolated from 7 CLL patients and treated for 24 h with 1 μM PAPTP. Kv1.3 expression, reported as mean fluorescence intensity (MFI, arbitrary units), is represented as a function of the viability of CLL B cells, estimated as the percentage of living cells (compared to non-treated ones) after treatment. The statistical relationship is assessed with Pearson’s correlation coefficient. Inset: Chemical structure of PAPTP. **b** Mitochondrial ROS production and mitochondrial membrane potential were measured, respectively, by MITOSOX (green lines) and TMRM (purple lines) fluorescence from ex vivo CLL B cells, isolated from 6 CLL patients, treated with 1 μM PAPTP or left untreated. The percentage of the initial values of MITOSOX and TMRM signals are reported as a function of treatment time and are shown as mean ± SD. Statistical significance of differences was assessed with the Multiple unpaired t test. Inset: Cytochrome c levels in the cytoplasm were evaluated by western blotting from ex vivo human CLL B cells treated with DMSO or PAPTP 1 μM (25 μg protein/lane). β-actin was chosen as cytoplasmic marker and is shown as well. **c** CLL B cells mortality was assessed after 24 h. Leukemic cells from 6 patients were cultured with allogeneic mesenchymal stromal cells (MSCs) as in [7] and treated with DMSO or PAPTP 1 μM. Data are shown as mean ± SEM. Statistical significance of differences was assessed with the Wilcoxon test. **d** Kv1.3 expression in CD19^+^CD5^−^ and CD19^+^CD5^+^ cells from peripheral blood of Eμ-*TCL1* mice was assessed by flow cytometry. Kv1.3 expression is reported as mean fluorescence intensity (MFI, arbitrary units). Data were obtained from 31 individual animals and are shown as mean ± SEM. Statistical significance of differences was assessed with the Wilcoxon test. **e** A representative fluorescein isothiocyanate (FITC) fluorescence distribution of the populations shown in panel d. **f** Percentage of CD19^+^ dead cells, estimated with Annexin V staining, was assessed by flow cytometry on ex vivo blood cells (isolated from WT and Eμ-*TCL1* mice), treated with DMSO or 5 μM PAPTP. Data were obtained from 4 independent experiments and are shown as mean ± SEM. Statistical significance of differences was assessed with the Mann–Whitney test

Kv1.3 is more expressed in human CLL leukemic B cells compared to B cells from healthy subjects [21]. Here we show that Kv1.3 expression in human primary CLL B cells correlates with sensitivity of the neoplastic cells to the mitochondria-targeted inhibitor PAPTP (1 μM) that acts on the mitochondrial counterpart of Kv1.3 (Fig. 1a). Treatment of CD5^+^CD19^+^ CLL B cells with PAPTP triggers an increase of mitochondrial reactive oxygen species (ROS) release (Fig. 1b) as well as depolarization of the mitochondrial membrane potential and cytochrome c release (Fig. 1b), in agreement with the general mechanism of action of PAPTP mentioned above [29]. Importantly, PAPTP was able to trigger death also when the cells were co-cultured with mesenchymal stromal cells, known to increase resistance to apoptotic stimuli (percentage of dead cells; alone: 24 ± 2, PAPTP: 75 ± 5; Fig. 1c).

These observations prompted us to test the effect of PAPTP also in vivo, in the widely used Eμ-*TCL1* mouse model. Since no information is available regarding the expression of K^+^ channels in CD5^+^CD19^+^ CLL B cells in this model, we first evaluated the expression level of Kv1.3 using a FITC-labelled antibody against an extracellular epitope of the protein (Fig. 1d, e), as described e.g. in [21]. We separately evaluated by flow cytometry the fluorescence signal from CD19^+^CD5^−^ healthy B cells and CD19^+^CD5^+^ CLL B cells isolated from the peripheral blood of 8–13-month-old Eμ-*TCL1* mice (see Supplementary Fig. 1 for disease progression). As illustrated in Fig. 1d, the expression levels of Kv1.3 were consistently 1.77-times higher in pathological (CD19^+^CD5^+^; 446 ± 17 mean fluorescence intensity, a.u., *n* = 31) cells than in healthy B lymphocytes (CD19^+^CD5^−^; 252 ± 10, *n* = 31, *p* < 0.0001).

We have previously shown that the mitochondria-targeted Kv1.3 inhibitor PAPTP is able to selectively kill cancer cells [29], due to synergy between inhibition of mtKv1.3 – highly expressed in neoplastic cells –leading to massive ROS production and the high basal levels of ROS [29, 47] typical of many types of cancer cells [48]; thus, PAPTP kills neoplastic cells by inducing strong oxidative stress above a critical threshold. Since expression of Kv1.3 in the plasma membrane positively correlates with the expression of the channel in the mitochondria, as observed in several cell types [32, 49], we next tested the effect of PAPTP on mouse CLL B cells. In accordance with the results obtained on primary human CLL B cells, 5 μM PAPTP induced apoptosis and killed 80% of the neoplastic cells from Eμ-*TCL1* mice within 24 h, without significantly affecting healthy B cells from WT ones, compared to a treatment with DMSO for the same time (percentage of dead cells; WT + DMSO: 22 ± 3; WT + PAPTP: 30 ± 1; Eμ-*TCL1* + DMSO: 25 ± 5; Eμ-*TCL1* + PAPTP: 78 ± 1; *n* = 4 for each group; Fig. 1f).

### PAPTP significantly ameliorates disease progression in Eμ-*TCL1* mice

**Fig. 2:**
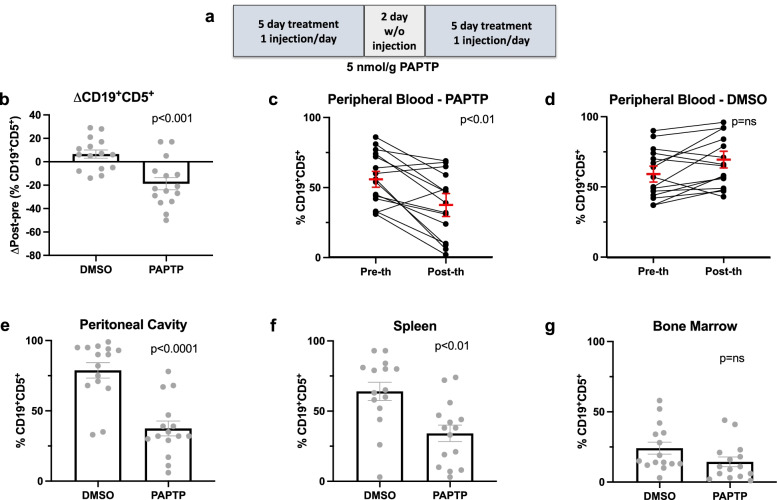
PAPTP-induced pathological CLL cell death in Eμ-*TCL1* mice. **a** Scheme representing the adopted protocol for PAPTP treatment: mice were injected once a day for 5 consecutive days; after 2 days of recovery, mice were subjected again to 5 injections, once a day for 5 consecutive days. **b-d** The percentage of B (CD19^+^CD5^+^) CLL cells was assessed by flow cytometry on ex vivo peripheral blood cells from Eμ-*TCL1* mice before and after intraperitoneal administration of DMSO or 5 nmol/g PAPTP. Data were obtained from 15 independent experiments for each condition. Statistical significance of differences was assessed with the Mann–Whitney test. Average differences between the post- and pre-treatment percentages of CD19^+^CD5^+^ cells are reported for each substance (**b**), as well as individual percentages before and after treatment (**c-d**). Statistical significance of differences was assessed with the Mann–Whitney test. **e-g** Percentages of CD19^+^CD5^+^ cells were assessed by flow cytometry on ex vivo cells from the peritoneal cavity (**e**), spleen (**f**), and bone marrow (**g**) of Eμ-*TCL1* mice after i.p. administration of DMSO (or PAPTP)

Given the strong, selective effect observed ex vivo, we then tested PAPTP in Eμ-*TCL1* mice in vivo CLL in humans is characterized by clonal expansion of small, mature-looking CD19^+^CD5^+^ B cells and by their accumulation in the blood, bone marrow, and lymphoid organs; we thus assessed the effect of PAPTP in vivo in various body districts and tissues in Eμ-*TCL1* mice where the same CD5^+^ B cell clone localizes to bone marrow (BM), spleen, peritoneal cavity and peripheral blood (PB) [36]. In addition, spleen size and histology were investigated as well, being splenomegaly and significant spleen hyperplasia a recurrent feature in 8–13-month-old Eμ-*TCL1* mice [34]. At the age of 8–13 months, peripheral blood samples were collected from Eμ-*TCL1* mice in order to confirm both surface antigen expression and clonality. Cells were stained with antibodies specific for murine CD45, CD5, CD19, and CD3 and evaluated by FC (gating strategy is shown in supplementary Fig. 2). Eμ-*TCL1* mice showing a percentage of pathological cells on the total lymphocyte number between 40 and 90% were treated by intraperitoneal injection (i.p.) of either DMSO (control) or 5 nmol/g PAPTP for 5 days every week, for 2 weeks (Fig. 2a). The dose of PAPTP utilized in this work has been previously demonstrated to be well tolerated by mice in melanoma and pancreatic adenocarcinoma orthotopic xenograft models [29]. Accordingly, mice did not show any sign of adverse effects or toxicity in the set of experiments performed in the present study. We then determined by FACS analysis the percentage of CD19^+^CD5^+^ cells in the PB of each Eμ-*TCL1* mouse before and after treating the animals with either the vehicle or PAPTP. Figure 2b shows the difference in the percentage of pathological CD19^+^CD5^+^ cells in the PB before and after DMSO (6.5 ± 3.5, *n* = 15) or PAPTP (− 18.7 ± 5.2, *n* = 15, *p* < 0.001) administration; the variation of pathological lymphocytes is expressed as the difference between the percentage of pathological B (CD19^+^CD5^+^) on total lymphocyte number at the start and at the endpoint. The same data are represented also as the variation of leukemic cells (pre vs post therapy) in each single PAPTP-treated animal (pre: 56.7 ± 4.8, post: 37.1 ± 6.0, *n* = 15, *p* < 0.01, Fig. 2c) and in DMSO-treated mouse (pre: 59.4 ± 4.5, post: 66.9 ± 4.6, *n* = 15, Fig. 2d). Of note, in four mice the percentage of pathological cells approached zero after the treatment and out of 15 mice treated with PAPTP, only in one there was progression of CLL (Fig. 2c) while in two other mice the percentage of neoplastic cells in the PB compared to pre-therapy was stable. So altogether in 14 mice out of 15 with distinct CLL B clones, the treatment had a beneficial effect. Similar results were obtained when assessing the progression of the disease in the peritoneal cavity (Fig. 2e, percentage of CD19^+^CD5^+^ on total lymphocytes; DMSO: 78.8 ± 5.5 vs PAPTP: 37.5 ± 5, *n* = 15 per group, *p* < 0.0001) and the spleen (Fig. 2f, *n* = 15 per group; DMSO: 64.1 ± 6.5 vs PAPTP: 34.1 ± 5.9, p < 0.01). A reduction of 50% of the percentage of pathological cells on the total lymphocyte number was detectable in both districts. The same tendency could be observed for BM; however, the difference was not statistically significant (Fig. 2g).

**Fig. 3:**
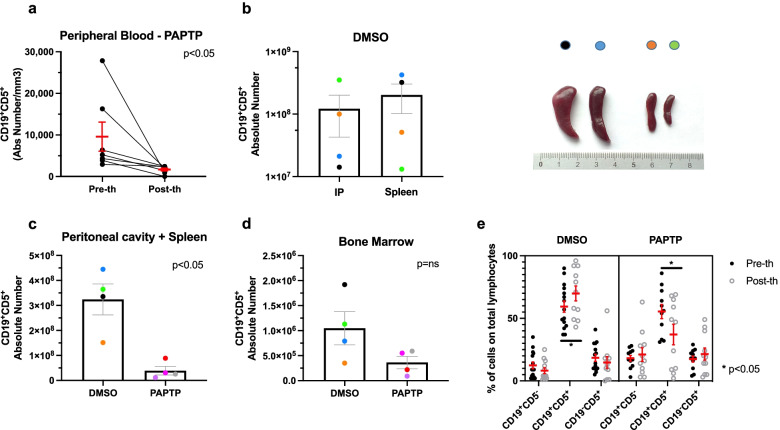
PAPTP reduces the absolute number of pathological CLL cell death in different body districts Eμ-*TCL1* mice. **a** The absolute number of B (CD19^+^CD5^+^) CLL cells was assessed by flow cytometry on ex vivo cells from peripheral blood from Eμ-*TCL1* mice after intraperitoneal administration of 5 nmol/g PAPTP as in Fig. 2 (*n* = 7). Statistical significance of differences were assessed with the Wilcoxon test. **b** The absolute number of B (CD19^+^CD5^+^) CLL cells was assessed by flow cytometry on ex vivo cells from peritoneal cavity and spleen from Eμ-*TCL1* mice after intraperitoneal administration of DMSO. Please note the logarithmic scale in y axis. Each mouse is indicated with a different color (*n* = 4). On the right, images of the spleens of the four examined animals are shown. In **c** leukemia burden is estimated by the sum of the absolute numbers of CLL B cells from peritoneal cavity and spleen in 8 animals (*n* = 4 for DMSO treatment, *n *= 4 for PAPTP treatment, as in Fig. 2a). Statistical significance of differences was assessed with the Mann–Whitney test. **d** as in c) for bone marrow is shown. **e** Percentages of healthy (CD19^+^CD5^−^) and CLL (CD19^+^CD5^+^) B cells, as well as of T (CD19^−^CD5^+^) cells, were assessed by flow cytometry on ex vivo PB cells from Eμ-*TCL1* mice before and after i.p. administration of DMSO or 5 nmol/g PAPTP. Mice were assigned to each group on the basis of the percentage of CD19^+^CD5^+^ cells, to minimize the difference in the distribution between the two groups; the absence of significant differences between the other cellular subtypes was assessed with the Mann–Whitney test. The relative abundance of each cellular subtype on the total lymphocytic number is reported for each condition. Data were obtained from 11 independent experiments, for each condition, as reported in Fig. 2b-d. Statistical significance of differences was assessed with the Mann–Whitney test

To further assess the leukemia burden, we performed another set of experiment, where the absolute number of CD19^+^CD5^+^ cells was determined in the sites of disease involvement, as shown in Fig. 3. Notably, in the peripheral blood the effect of PAPTP was highly significant as it caused an almost six-fold decrease of CLL B cells (Fig. 3a) (*n* = 7, pre-therapy: 9562 ± 3494 CD5^+^CD19^+^ /mm^3^ blood; PAPTP: 1665 ± 345 CD5^+^CD19^+^ /mm^3^). Next, we examined the distribution of the pathologic cells within CLL-relevant disease sites in a cohort of DMSO-treated mice, namely the spleen and peritoneal cavity (PC). We noticed that different clones of CLL B cells growing in individual Eμ-*TCL1* mice distribute differently in these sites (*n* = 4; Fig. 3b): in the graph, each mouse is indicated with a different color to highlight that the absolute number of CLL B cells reciprocally correlate between spleen and the peritoneal cavity and the spleen size (see photo in Fig. 3b) roughly correlated with the absolute number of pathologic cells, as expected (please note that y axis is on a logarithmic scale). The leukemia burden, estimated thus by the sum of pathological cells in peritoneal cavity and spleen, strongly decreased (by 88%) upon treatment with PAPTP (Fig. 3c, DMSO: 3.24 × 10^8^ ± 6.20 × 10^7^ vs PAPTP: 3.9 × 10^7^ ± 1.72 × 10^6^, *n *= 4, < 0.05), while the decrease did not reach significant levels in the bone marrow (Fig. 3d).

**Fig. 4:**
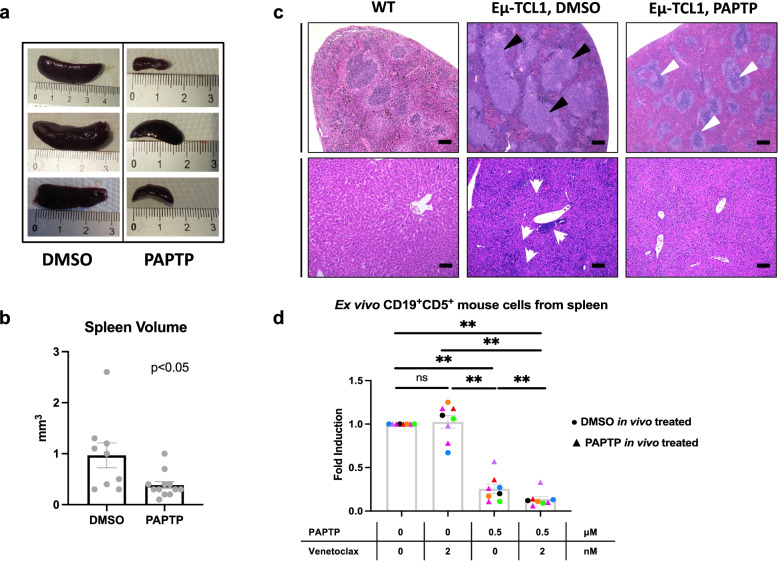
PAPTP reduces splenomegaly and ameliorates tissue morphology in Eμ-*TCL1* mice. **a** Representative spleens from Eμ-*TCL1* mice treated with DMSO or PAPTP. **b** Spleen volume of Eμ-*TCL1* mice treated with DMSO or 5 nmol/g PAPTP. Data were obtained from 9 mice treated with DMSO and 12 mice treated with PAPTP. **c** Histological sections from spleen and liver samples of WT mice and from Eμ-*TCL1* mice treated with DMSO or PAPTP were stained with hematoxylin and eosin. Spleen sections from Eμ-*TCL1* mice treated with DMSO show large nodular lymphoid aggregates (black triangles), that are not visible in sections from mice treated with PAPTP, where normal follicles can be observed (white triangles). Liver sections from mice treated with DMSO disclosed a lymphoid infiltrate akin to that observed in the spleens, with small portal, intra-lobular and sinusoidal/interstitial growth pattern and occasional lymphoid aggregates of neoplastic cells (white arrowheads, please note a big neoplastic lymphocytic nodule in the center of the image). Intra-hepatic lymphocytes were scant to absent in both PAPTP-treated and control livers. Bar corresponds to 100 μm in all images. **d** Ex vivo spleen cells were extracted from the same Eμ-*TCL1* mice injected with DMSO or with 5 nmol/g PAPTP shown in Fig. 3. The expression level of the channel did not change: mean fluorescence intensity was (398.75 ± 73.39 (SD) (*n* = 4) before treatment versus 423.25 ± 95.66 (SD) (*n* = 4) after treatment, *p* = 0.69, t-test (not shown). The cells were then treated with 0.5 μM PAPTP, 2 nM Venetoclax or with both, for 20 h. Percentages of dead pathological B (CD19^+^CD5^+^) CLL cells were assessed by flow cytometry (via Annexin staining) for each condition. Note that the color code is the same of that used in Fig. 3. Data are shown as fold induction of the control (untreated) and mean ± SEM values are depicted by histograms. Statistical significance of differences was assessed with the Wilcoxon test

Importantly, PAPTP induced death of pathological CLL selectively in Eμ-*TCL1* mice (Fig. 3e). For both untreated and treated animals, the percentage of healthy B (CD19^+^CD5^−^) and T (CD19^−^CD5^+^; confirmed with CD3^+^) cells in the PB did not significantly change (Fig. 3e). PAPTP exerted a huge beneficial effect on spleen volume and histological features as well: the spleen volume, in particular, was more than 2-fold smaller compared to that of DMSO-treated mice, approaching a value comparable to the normal one (Fig. 4a and b, DMSO: 1.0 ± 0.2 mm^3^, *n* = 9; PAPTP: 0.4 ± 0.1 mm^3^, *n* = 12, *p* < 0.05). In addition, histological evaluation of the spleen from vehicle-treated and PAPTP-treated Eμ-*TCL1* mice disclosed considerable differences. The analysis was extended to liver, as infiltration in these organs has been documented in Eμ-*TCL1* mice [41]. Specifically, in the DMSO-treated group multiple lymphoid aggregates were detected, with intra-acinar, perivenular and intra-sinusoidal distribution; neoplastic lymphocytes were small with round nuclei and clumped chromatin, consistent with CLL/small lymphocytic lymphoma (SLL) morphology (Fig. 4c). A similar lymphoid infiltrate was also documented in the splenic white pulp, which was effaced and expanded by large, confluent nodules of monomorphous small lymphocytes. These findings are consistent with those observed in Eμ-*TCL1* mice [33] and in patients with CLL [50]. Such infiltrate was not documented in the liver and spleen of PAPTP-treated mice, that displayed a normal tissue architecture (Fig. 4c). Altered histology was observable in the spleen of Eμ-*TCL1* mice independently of spleen size: small spleen in the DMSO-treated animal displayed all the above characteristics of CLL spleen, while a spleen of the same size obtained after treatment with PAPTP showed histological features close to normal WT spleen (Supplementary Fig. 3). It is of note that when we isolated pathological B cells from PAPTP-treated animals and then treated them in ex vivo setting, they still responded to PAPTP, suggesting that resistance did not develop during the 2-week treatment (Fig. 4d and Supplementary Fig. 4).

Altogether, a comparison of vehicle versus compound effect in different Eμ-*TCL1* animals bearing unique clones (as in the case of humans), allowed us to conclude that PAPTP administered at a low, non-toxic concentration significantly reduced the percentage and absolute number of pathological cells in CLL-relevant districts, even in animals that were in a very advanced stage of the disease (up to 90% of pathological cells in the PB).

### Lack of significant effects of TRAM-34 in Eμ-*TCL1* mice

**Fig. 5:**
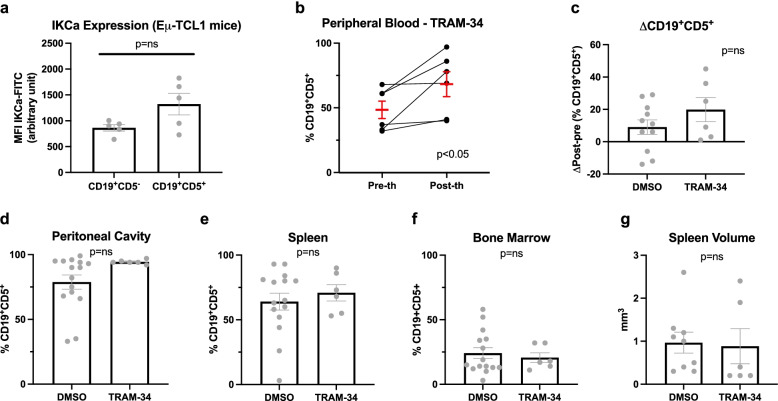
IKCa inhibition does not induce CLL cell death in Eμ-*TCL1* mice. **a** IK_Ca_ expression in CD19^+^CD5^−^ and CD19^+^CD5^+^ cells from peripheral blood of Eμ-TCL1 mice was assessed by flow cytometry. IK_Ca_ expression is reported as mean fluorescence intensity (MFI, arbitrary units). Data were obtained from 5 independent experiments and are shown as mean ± SEM. Statistical significance of differences was assessed with the Mann–Whitney test. **b-c** Percentage of CD19^+^CD5^+^ cells was assessed by flow cytometry on ex vivo peripheral blood cells from Eμ-*TCL1* mice before and after i.p administration of DMSO or 10 nmol/g TRAM-34 (**b**). Data were obtained from 15 independent experiments with DMSO and 6 with TRAM-34. As the quantity of DMSO injected with PAPTP or with TRAM-34 was the same, data from all mice that received DMSO are pooled (*n* = 15, shown in Fig. 2d). Statistical significance of differences was assessed with the Wilcoxon test. **c** Differences between the post- and pre-treatment percentages of CD19^+^CD5^+^ cells are reported for the experiments shown in **b**. Statistical significance of differences was assessed with the Mann–Whitney test. **d-g** Percentages of CD19^+^CD5^+^ cells were assessed by flow cytometry on ex vivo cells from peritoneal cavity (**d**), spleen (**e**), and bone marrow (**f**) of Eμ-*TCL1* mice before and after i.p. administration of DMSO or TRAM-34. Statistical significance of differences was assessed with the Mann–Whitney test. **g** Spleen volume of the mice reported above was measured by hydrostatic weighing. Statistical significance of differences was assessed with the Mann–Whitney test

A previous work reported that the IKCa inhibitor TRAM-34 decreased Ki-67-expression in human CLL cells ex vivo by 55% [20]. The channel protein was detectable in the mitochondria-enriched fractions of human CLL cells and these cells were sensitive to TRAM-34 which was able to trigger apoptosis at 10 μM concentration (Supplementary Fig. 4). In order to assess whether this ability may translate into an improvement in terms of immunological and clinical outcome, TRAM-34 was tested in Eμ-*TCL1* mice in vivo. Using FITC-labelled anti-IKCa antibody, we first confirmed that in pathological cells of Eμ-*TCL1* mice the channel shows somewhat higher expression compared to normal lymphocytes (Fig. 5a). While expression of Ki-67 was not significantly altered upon incubation of CLL B cells isolated from spleen with TRAM-34, the proportion of viable cells decreased upon incubation with 10 μM concentration of the drug (Supplementary Fig. 5). Administration of TRAM-34 at 10 nmol/g, with the same treatment protocol adopted for PAPTP, was well tolerated by the mice. Estimating the total blood volume by the formula: blood volume (ml) = 0.06 x body weight (g) + 0.77 [51], if all the drug immediately reached the bloodstream, administration of 300 nmols of TRAM-34 would result in a concentration of approximately 120 μM, a value ten times higher than that previously shown to reduce proliferation of activated CLL B cells ex vivo [20] and to reduce cell viability (Supplementary Figs. 4 and 5). Nonetheless, i.p. injection of 10 nmol/g TRAM-34 for ten days did not improve disease control, measured as the percentage of pathological cells upon treatments (Fig. 5b) or as the difference in the percentage of CD19^+^CD5^+^ cells in the PB (Fig. 5c). A tendency to an increase of the percentage of CD19^+^CD5^+^ cells was observed in the peritoneal cavity (Fig. 5d) as well as in the spleen (Fig. 5e) but not in the bone marrow (Fig. 5f). In addition, no significant change in spleen volume occurred (Fig. 5g).

### Comparison of the effects of Venetoclax and PAPTP on CLL B cells

The aforementioned results strongly indicate that, while TRAM-34 does not exert a beneficial effect in this mouse model in vivo, PAPTP is able to significantly reduce CLL progression even in animals with advanced-stage disease. Therefore, we aimed at investigating whether the benefit produced by PAPTP could resemble that obtained by administration of Venetoclax (ABT-199), a pro-apoptotic agent acting at the level of the outer mitochondrial membrane. First, efficacy of PAPTP versus Venetoclax (ABT-199) in murine CLL B cells was investigated. We treated ex vivo CLL B cells isolated from the spleen of 8 Eμ-*TCL1* mice, with either Venetoclax or PAPTP or both together (Fig. 4d). Isolated pathological B cells from PAPTP-treated animals still responded to PAPTP, but Venetoclax did not trigger CLL B-cell death (Fig. 4d), in agreement with previous reports showing that spleen-derived cells are resistant to even 5 nM Venetoclax [52], probably due to dependence of these cells on anti-apoptotic proteins MCL1 (myeloid cell leukemia sequence 1) and BFL1 (BCL2-related protein A1). In accordance, in two different studies, Venetoclax did not exert any positive effect on leukemia burden in Eμ-*TCL1* mice model [39, 52].

**Fig. 6:**
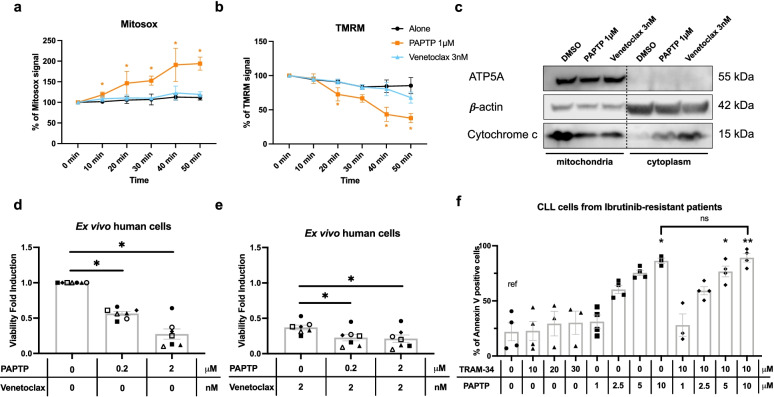
Comparison of PAPTP and Venetoclax effects in human B CLL cells ex vivo*.* **a-b** Mitochondrial ROS production **(a)** and mitochondrial membrane potential **(b)** were measured, respectively, by MITOSOX (**a**) and TMRM (**b**) fluorescence from ex vivo CLL B cells, isolated from 3 CLL patients, either treated with 3 nM Venetoclax or untreated. The percentage of the initial values of MITOSOX and TMRM signals are reported as a function of treatment time and are shown as mean ± SD. For comparison of the kinetics induced by Venetoclax and PAPTP, data shown in Fig. 1b for PAPTP are indicated also here. Statistical significance of differences was assessed with Multiple unpaired t-test. **c** Cytochrome c levels in mitochondria and cytoplasm were evaluated by western blotting from ex vivo human CLL B cells treated with DMSO or 3 nM Venetoclax (25 μg protein/lane). ATP5A and β-actin were chosen as mitochondrial and cytoplasmic markers and are shown as well. **d**, **e** Ex vivo peripheral blood cells, isolated from 7 CLL patients were treated with 0.2 μM or 2 μM PAPTP alone (**d**) or in combination with 2 nM Venetoclax (**e**). The percentage of CD19^+^CD5^+^ dead cells, estimated with Annexin V staining, was assessed by flow cytometry for each condition and is shown as fold induction of the untreated control (alone) and mean ± SEM values are depicted by histograms. Statistical significance of differences was assessed with the Wilcoxon test. **f** Percentage of dead cells, based on Annexin V staining, was assessed by flow cytometry on cultured ex vivo B CLL cells isolated from ibrutinib-resistant patients. Cells were treated with different concentrations of TRAM-34 or with different concentrations of PAPTP, alone or in combination, as indicated. Data were obtained from 4 patients and are represented as mean ± SEM. Statistical significance of group differences compared to the untreated one were assessed with the Kruskal-Wallis test (*** *p* ≤ 0.001); statistical significance of differences between individual groups were assessed with the Mann–Whitney test

Thus, we tested the effects of PAPTP in comparison to Venetoclax at their respective half maximal effective concentration (EC_50_) values for apoptosis induction on human ex vivo cells, in particular on mitochondrial parameters and cytochrome c release (Fig. 6a-c). While PAPTP is able to trigger a rapid mitochondrial ROS production and inner membrane depolarization, within the examined time frame Venetoclax has no significant effect. PAPTP did not alter these parameters in B cells isolated from healthy subjects (Supplementary Fig. 6), in accordance with the lack of death induction. The lack of effect by Venetoclax is in agreement with previous reports that do not show an instantaneous mitochondrial membrane depolarization using this drug (see e.g. [53, 54]. Finally, using concentrations of PAPTP and Venetoclax close to the respective EC_50_ values (200 nM and 2 nM, respectively), we tested whether ex vivo human CLL B cells that survive Venetoclax treatment are still sensitive to PAPTP. Venetoclax acts on the outer-membrane located anti-apoptotic protein Bcl-2, while PAPTP has a different mode of action: it closes the mitochondrial Kv1.3 channel located in the inner mitochondrial membrane, thereby triggering a rapid increase in ROS production (Fig. 6a) and depolarization of the mitochondrial inner membrane due to permeability transition pore opening (Fig. 6b), finally leading to cytochrome c release (Fig. 6c). Figure 6 d and e show that as expected, due to the different mode of action, PAPTP is able to enhance the effect of Venetoclax (both applied at sub-μM doses), suggesting that PAPTP might be useful for Venetoclax-resistant patients. Finally, when added to primary cells isolated from ibrutinib-resistant patients, PAPTP, but not TRAM-34, was able to kill these cells (Fig. 6f). A higher concentration of PAPTP was required compared to cells obtained from non-treated patients, likely due to anti-oxidant system upregulation in ibrutinib-resistant cells (see e.g. [55]). Indeed, increased glutathione level were associated with insensitivity to inhibition of BCR survival signaling also in diffuse large B cell lymphoma [56].

## Discussion

The present study identifies PAPTP, a mitochondriotropic compound acting on mtKv1.3 for which pharmacokinetics and lack of toxicity have previously been reported [29], as a promising drug against CLL. The in vivo data reported here show for the first time a potent beneficial effect of PAPTP in Eμ-*TCL1* mice that represents an established animal model of aggressive CLL. In our in vivo experiments we used individual Eμ-*TCL1* mice with overt pathological state which represents a situation that is more closely resembling the one encountered in the clinic: each patient harbors specific B-CLL clones, exactly as the distinct individual Eμ-*TCL1* mice do (e.g. [57]).

Our and others’ previous works showed that the *Shaker* type potassium channel Kv1.3 is overexpressed in several types of cancer, including CLL [21, 22, 29, 32, 58–60]. Ex vivo human CLL B cells, but not the residual T lymphocytes of the same patients, were killed by mtKv1.3-targeting agents even when anti-apoptotic Bcl-2 was overexpressed and the tumor suppressor p53 was mutated [32]. Several drugs directly targeting proteins of the inner mitochondrial membrane are characterized by their ability to bypass upstream events necessary for triggering intrinsic apoptosis (for recent review see e.g. [61]). This may explain our finding that PAPTP was so effective, even on CLL B cells from ibrutinib-resistant patients. Ibrutinib, to date is among the most effective therapies in CLL along with Venetoclax, but as mentioned above, resistance may develop (e.g. [62]). While Venetoclax targets the cell death signaling pathways mediated by the outer mitochondrial membrane, ibrutinib acts at the plasma membrane via the B cell receptor (BCR). Conversely, mitoKv1.3 channels are located in the inner mitochondrial membrane, where they affect cell death signaling independently of the apoptotic regulatory machinery of the OMM and independently of the BCR pathway.

Even though ion channels are druggable targets, to our knowledge no studies addressed the in vivo effects of highly specific ion channel modulators on CLL disease progression in an animal model to date. Besides Kv1.3 and IKCa, several other ion channels have been linked to CLL, at least in in vitro or ex vivo experiments. For example, HERG (human ether-a-go-go related gene, also called Kv11.1) was found to be upregulated in primary CLL B cells [58]. While its role has been elucidated in chronic myeloid leukemia (CML) cells (e.g. [63]) and in acute lymphoblastic leukemia [64], to our knowledge no studies addressing the mechanism linking HERG to CLL B lymphocyte proliferation or pharmacological studies targeting HERG in CLL B cells were performed. Polymorphism of the P2X7 receptor (P2X7R, P2X purinoceptor 7), a ligand-gated cation channel that mediates ATP-induced apoptotic death in haemopoietic and CLL cells, has been observed to correlate with clinical outcome [65]. Overexpression of P2X7R, promoted by downregulation of NLRP3 (NACHT, LRR and PYD domains-containing protein 3) inflammasome, correlates with chromosome 12 trisomy in CLL patients [66]. Interestingly, the HVCN1 (Hydrogen voltage-gated channel 1), the only mammalian voltage-gated proton channel, has been proposed to contribute to pathogenesis [67]: HVCN1 associates with the BCR and is required for optimal BCR signaling and redox control. Recent studies highlighted a possible role also for constitutive calcium entry-linked Orai and TRPC1 (transient receptor potential canonical) channels as well as the regulator STIM1 in pathogenesis [68]. While all these channels represent interesting pharmacological targets for possible treatment of CLL, the effects of their modulation by specific activators/inhibitors or monoclonal antibodies have been addressed in relatively few ex vivo studies, focusing mainly on proliferation.

Mitochondria play a crucial role in apoptosis that may represent a useful way to remove pathological CLL B cells. Pharmacological inhibition of a mitochondrial potassium channel causes a block of the depolarizing K^+^ influx into the matrix, down the electrochemical gradient, with the consequent hyperpolarization of the inner mitochondrial membrane [28]. This change in membrane potential, as well as the physical proximity of mtKv1.3 to Complex I of the respiratory chain [47], trigger enhanced ROS release that in turn opens the permeability transition pore. When this pore is open, depolarization as well as mitochondrial swelling occur, leading to rupture of the outer mitochondrial membrane and to cytochrome c release. Despite Kv1.3 being a depolarization-activated channel, it is functional at resting mitochondrial potential, since its inhibition changes this bioenergetic parameter [28]. Mitochondria also harbor a number of ion channels whose pharmacological targeting may alter mitochondrial metabolism, leading to cytochrome release and triggering of programmed cell death (for recent reviews see e.g. [26, 69]). For example, synthetic, membrane-penetrating peptides able to prevent the interaction of anti-apoptotic mitochondrial proteins with the mitochondrial outer membrane-located porin (also called voltage-dependent anion channel (VDAC)), were shown to activate apoptosis in CLL B cells, at least in vitro [70].

PAPTP kills different types of cancer cells, provided they express high levels of Kv1.3 and have an altered redox state and in particular an elevated basal ROS level, recognized as a factor interfacing intracellular signaling pathways to ensure cell survival [71]. PAPTP, by inhibiting mtKv1.3 substantially increase harmful ROS production [47] and drives the cells towards ROS-triggered apoptosis [29]. Such a strategy can be useful in general in the case of CLL pathology. In fact, prevalent glycolytic metabolism, along with reduced pentose-phosphate pathway-dependent antioxidant defense system, is an important characteristic of B cell tumors [72]. Stromal cells were shown to promote CLL B cell survival by aiding anti-oxidant glutathione synthesis in pathological cells, as demonstrated also in vivo in a seminal study, using the Eμ-*TCL1* mouse [73]. This model of CLL is indeed relevant also to assess the protective role of the stromal microenvironment for CLL cells upon treatment, since the *tcl-1* gene, encoding an AKT co-activator, is significantly upregulated in CLL B lymphocytes upon co-culturing with stromal cells [74]. A recent study tested the effect of auranofin, an oral gold-containing triethylphosphine used against rheumatoid arthritis and acting as an inhibitor of thioredoxin reductase. This study showed that auranofin was able to trigger lethal oxidative stress in CLL cells [40] and was therefore able to overcome apoptosis resistance mediated by protective stromal cells, even in Eμ-*TCL1* mice in vivo. Treatment with auronafin (10 mg/kg daily, corresponding to 15 nmol/g, 5 days per week for 2 weeks), reduced total leukemia cell burden in the peritoneal fluid by approximately 70%; unfortunately, the study did not address the effect of this drug on spleen, bone marrow and healthy cells. Altogether, the above studies, along with data presented here, point to the possibility of exploiting the sensitivity of CLL B cells to oxidative stress for therapeutic purposes. It is worth mentioning that PAPTP, besides killing pathologic cells, at lower, sub-lethal concentrations may also exert a beneficial effect against CLL since it was shown to reduce the bioenergetic efficiency of mitochondria by acting on mtKv1.3, and, as a consequence, triggering endoplasmic reticulum (ER) stress that ultimately leads to an inhibition of Wingless-related integration site (Wnt) signaling [75]. The importance of Wnt signaling for CLL has already been demonstrated [76].

The question arises of the observed lack of effects of TRAM-34 on disease progression in the Eμ-*TCL1* mice, even though this drug was able to reduce proliferation of human CLL B cells ex vivo [20] and we show that murine CLL cells are sensitive to TRAM-34. This drug, when injected i.p. (10 mg/kg, corresponding to 30 nmol/gbw), was shown to be able to quickly reach even the brain, as it passes the blood brain barrier and exerts a positive, protective effect in ischemic brain [77], indicating that the drug is stable in the body. However, being this drug highly hydrophobic, issues linked to pharmacokinetics and/or to an anti-apoptotic effect of the tumor microenvironment cannot be excluded as the possible cause of the lack of decrease of leukemic cells in our animals, where the drug was injected i.p. In vitro, TRAM-34 has been shown to induce apoptosis in several types of cancer cells. For example in murine GL261 and human U87MG malignant glioma cells, this drug alone increased the percentage of Annexin V-positive cells at 5 μM concentration, although it did not exert the same action in patient-derived GBM18 cells [78]. In the same study, intracranial inoculation of 120 mg/kg/day (corresponding to 360 nmol/g) significantly increased survival of mice with glioblastoma [78]. Thus, a higher, optimized in vivo dosage/administration in the context of the *TCL1* mouse model might be warranted in future studies. In other studies, TRAM-34 alone was unable to trigger apoptosis of melanoma cell lines, but significantly enhanced the effect of vemurafenib even in cells that were resistant to this latter drug [79], in contrast to our observations where TRAM-34 was not effective on ibrutinib-resistant CLL cells. Altogether, further work is required to understand the factors that contribute to apoptosis induction by TRAM-34 in a cell type-dependent manner.

In summary, our work suggests that PAPTP might be a promising choice to specifically kill pathological B cells. Importantly, while many studies using the Eμ-*TCL1* mouse model reported a beneficial effect of various drugs administered at early age to these mice, PAPTP was able to significantly slow down disease progression even in mice with advanced state CLL. Since the onset and severity of the disease is variable in the *TCL1* genetic model (as illustrated in Supplementary Fig. 1), a meaningful long-term survival study is generally not performed using this model.

## Conclusion

Taken together, our data identify an ion channel, namely the mitochondrial Kv1.3 potassium channel, as a promising pharmacological target against aggressive CLL. We show a significant beneficial effect of the mitochondriotropic drug PAPTP that specifically targets this mitochondrial channel, in Eμ-*TCL1* mice, the widely used animal model of aggressive CLL. Importantly, the effect of the drug is not restricted to CLL B cells of the peripheral blood, but PAPTP is efficient in killing pathologic B cells also in other relevant body district, namely the spleen and the peritoneal cavity. While inducing apoptosis of CLL B cells that express high level of Kv1.3, PAPTP does not alter the survival of healthy B and T cells. In human cells from CLL patients, PAPTP triggers apoptosis even in cells obtained from ibrutinib-resistant patients and enhances the effect of Venetoclax. Thus, PAPTP might be useful against advanced-stage CLL and even in the case of chemoresistant forms of this disease.

## Supplementary Information

**Additional file 1: Supplementary Figure 1.** Kaplan-Meier Estimates. of the probability of developing disease during the life span of Eμ-*TCL1* mice. **Supplementary Figure 2.** Flow cytometric panels showing the gating strategy. **Supplementary Figure 3.** Histological features of spleens from Eμ-*TCL1* mice with no splenomegaly. **Supplementary Figure 4.** Residual CLL cells from PAPTP-treated Eμ-*TCL1* mice respond to PAPTP treatment in ex vivo setting. **Supplementary Figure 5.** Expression of KCa3.1 channel in mitochondria of human B-CLL cells and effect of TRAM-34 on death. **Supplementary Figure 6.** Effect of TRAM-34 on proliferation and survival of B-CLL cells from Eμ-*TCL1* mice. **Supplementary Figure 7.** PAPTP does not induce ROS release and mitochondrial depolarization in B lymphocytes of healthy subjects.

## Data Availability

All data generated or analysed during this study are included in this published article and its supplementary information files.

## Abbreviations

APC: Allophycocyanin
ATP: Adenosine Triphosphate
Bcl-2: B-cell lymphoma 2
BCR: B-cell receptor
BFL1: BCL2-related protein A1
BM: Bone marrow
BTK: Bruton’s tyrosine kinase
CD: Cluster of differentiation
CLL: Chronic lymphocytic leukemia
CML: Chronic myeloid leukemia
CXCR4: C-X-C motif chemokine receptor 4
DMSO: Dimethyl sulfoxide
EC_50_: Half maximal effective concentration
ER: Endoplasmic reticulum
FACS: Fluorescence-activated cell sorter
FC: Flow cytometry
FITC: Fluorescein isothiocyanate
HERG: Human ether-a-go-go related gene
HVCN1: Hydrogen voltage-gated channel 1
Ibrutinib: 1-[(3R)-3-[4-Amino-3-(4-phenoxyphenyl)-1H-pyrazolo[3,4-d]pyrimidin-1-yl]-1-piperidinyl]-2-propen-1-one
IKCa: Intermediate-conductance calcium-dependent potassium channel (KCa3.1)
MCL1: myeloid cell leukemia sequence 1
mitoKv1.3: Inner mitochondrial membrane-located Kv1.3 potassium channel
mitoSOX: Triphenylphosphonium-linked hydroethidium
NLRP3: NACHT, LRR and PYD domains-containing protein 3
P2X7R: P2X purinoceptor 7
PAP-1: 5-(4-Phenoxybutoxy)psoralen
PAPTP: 3-(4-(4-((7-oxo-7H-furo[3,2-g]benzopyran-4-yl)oxy)butoxy)phenyl)propyl)triphenyl phosphonium iodide
PB: Peripheral blood
PBS: Phosphate buffered saline
PC: Peritoneal cavity
PE: Phycoerythrin
ROS: Reactive oxygen species
SLL: Small lymphocytic lymphoma
STIM1: Stromal interaction molecule 1
TCL1: T cell leukemia/lymphoma 1A
TMRM: Tetramethylrhodamine
TRAM-34: 1-[(2-Chlorophenyl)diphenylmethyl]-1H-pyrazole
TRPC: Transient receptor potential canonical
VDAC: Voltage-dependent anion channel
Venetoclax: 4-[4-[[2-(4-chlorophenyl)-4,4-dimethylcyclohexen-1-yl]methyl]piperazin-1-yl]-N-[3-nitro-4-(oxan-4-ylmethylamino)phenyl]sulfonyl-2-(1H-pyrrolo[2,3-b]pyridin-5-yloxy)benzamide
Wnt: Wingless-related integration site
