# Supplementary material for: Pharmacological modulation of Kv1.3 potassium channel selectively triggers pathological B lymphocyte apoptosis in vivo in a genetic CLL model
Source: J Exp Clin Cancer Res. 2022 Feb 16;41:64. doi: 10.1186/s13046-022-02249-w (PMC8848658; doi:10.1186/s13046-022-02249-w)
Supplement: Supplementary file 1 — Additional file 1: Supplementary Figure 1. Kaplan-Meier Estimates. of the probability of developing disease during the life span of Eμ-TCL1 mice. Supplementary Figure 2. Flow cytometric panels showing the gating strategy. Supplementary Figure 3. Histological features of spleens from Eμ-TCL1 mice with no splenomegaly. Supplementary Figure 4. Residual CLL cells from PAPTP-treated Eμ-TCL1 mice respond to PAPTP treatment in ex vivo setting. Supplementary Figure 5. Expression of KCa3.1 channel in mitochondria of human B-CLL cells and effect of TRAM-34 on death. Supplementary Figure 6. Effect of TRAM-34 on proliferation and survival of B-CLL cells from Eμ-TCL1 mice. Supplementary Figure 7. PAPTP does not induce ROS release and mitochondrial depolarization in B lymphocytes of healthy subjects. [file 13046_2022_2249_MOESM1_ESM.pdf]

Pharmacological modulation of Kv1.3 potassium channel selectively triggers pathological B lymphocyte apoptosis *in vivo* in a genetic CLL model

Severin et al, Supplementary Information

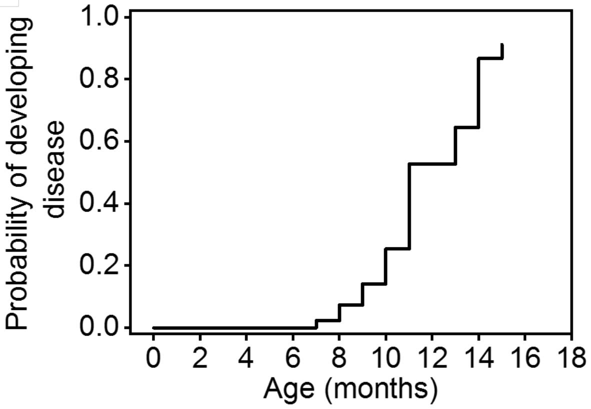

Supplementary Figure 1. Kaplan-Meier Estimates of the probability of developing disease during the life span of Eµ-*TCL1* mice. Animals that developed the disease (above 40% pathologic B cells) were considered (n=52).

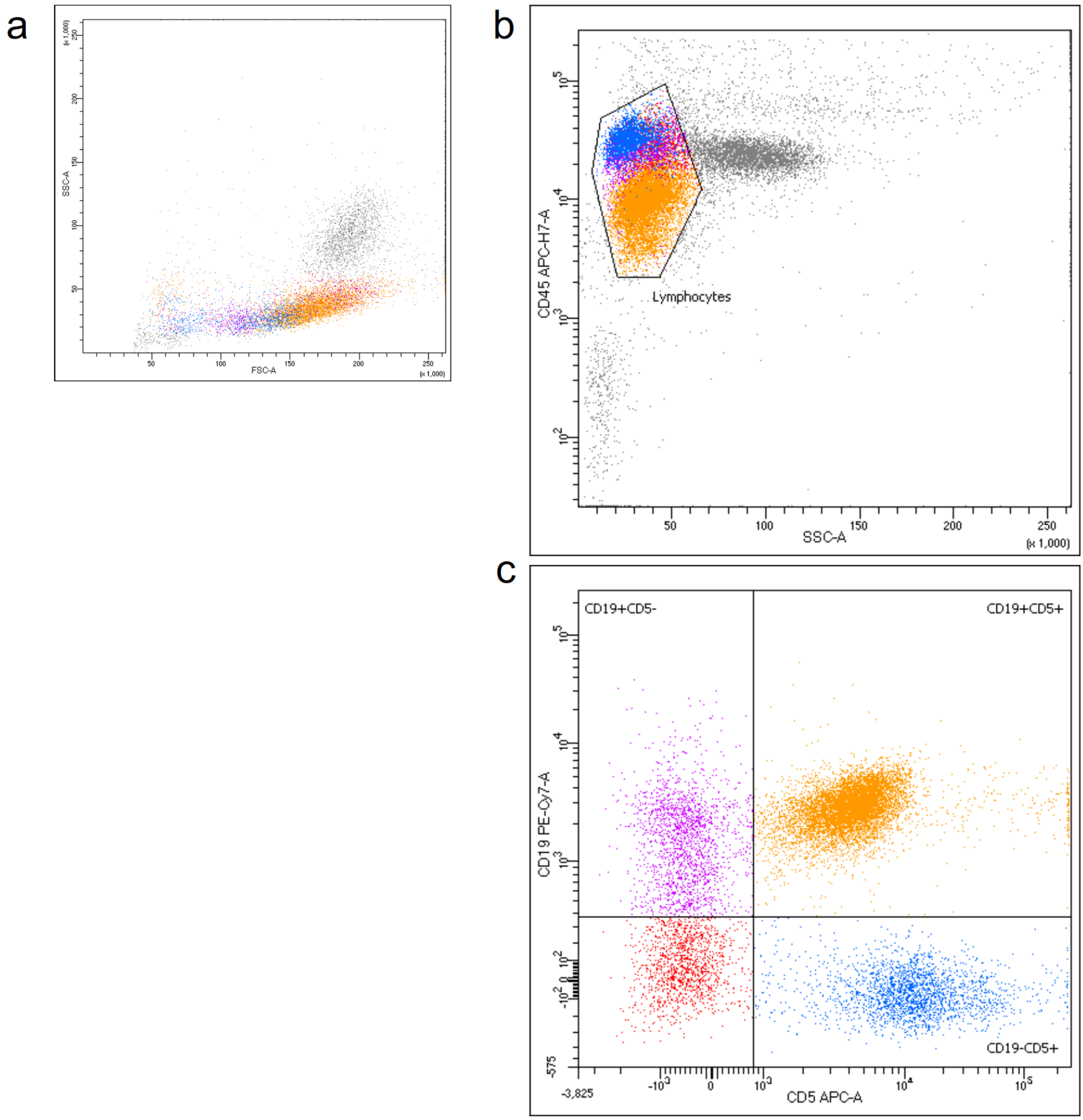

Supplementary Figure 2 – Flow cytometric panels showing the gating strategy. a) Representative morphological dot plot (forward and side light scatter) of peripheral blood (PB) cells from a Eμ-*TCL1* mouse. **b)** PB cells from Eμ-*TCL1* mice were gated on lymphocytes, based on side light scatter and CD45 staining, and **c)** analyzed for CD5 and CD19 expression.

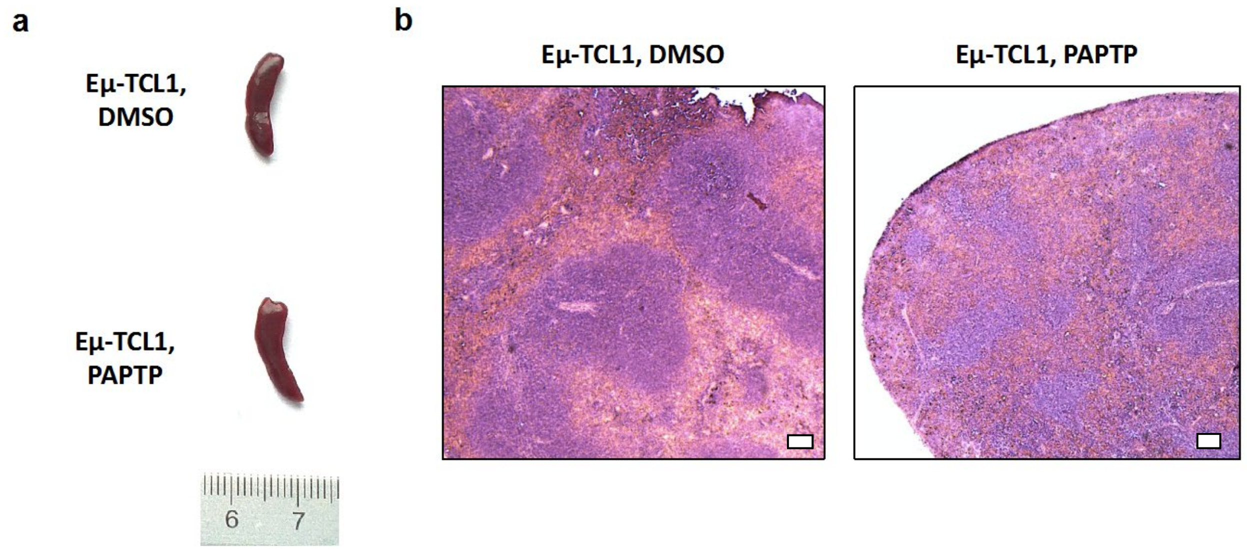

Supplementary Figure 3 – Histological features of spleens from Eμ-*TCL1* mice with no splenomegaly. Spleens from two Eμ-*TCL1* mice, treated respectively with DMSO or PAPTP as in Figure 2a, with no marked splenomegaly are shown. The spleen from the mouse treated with DMSO shows marked expansion of the white pulp compared to PAPTP-treated spleen. White pulp lymphocytes are small to medium sized with clumped chromatin, consistent with CLL/SLL cytology. The representative images shown here refer to the mice indicated with orange in Figure 3b (DMSO-treated) and to the "magenta" mice (PAPTP-treated). White bars correspond to 100 μm.

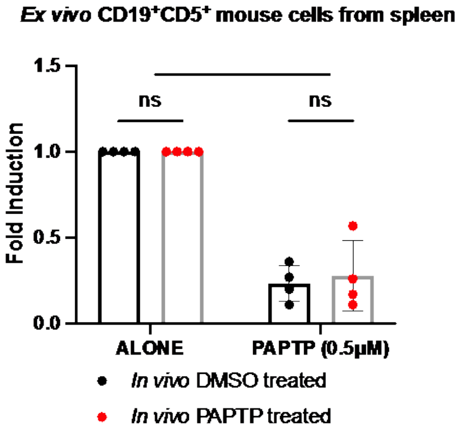

Supplementary Figure 4 – Residual CLL cells from PAPTP-treated Eμ-*TCL1* mice respond to PAPTP treatment in *ex-vivo* setting. Data shown refer to those reported in Figure 4e for cells isolated from the spleen of PAPTP or DMSO-treated animals and then incubated with PAPTP.

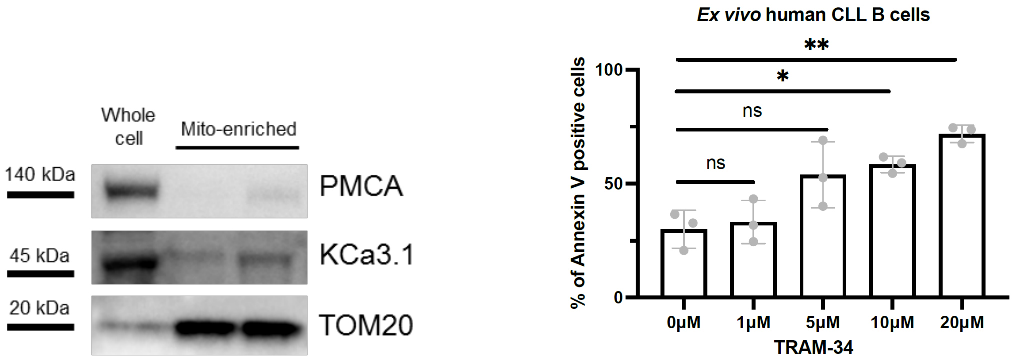

Supplementary Figure 5 – Expression of KCa3.1 channel in mitochondria of human B-CLL cells and effect of TRAM-34 on death. 25 µg of whole cell lysates and mitochondria-enriched fractions were loaded on an SDS-PAGE and blotted with the indicated antibodies. TOM20 is a marker of mitochondria, while PMCA (plasma membrane Calcium ATP-ase) is a marker of the plasma membrane. IKCa (KCa3.1) was detected at the expected molecular weight. The blot shown is representative of 4 blots obtained with the cells of different patients. Right panel: Cell death was assessed as described in the materials and methods section (24 h incubation).

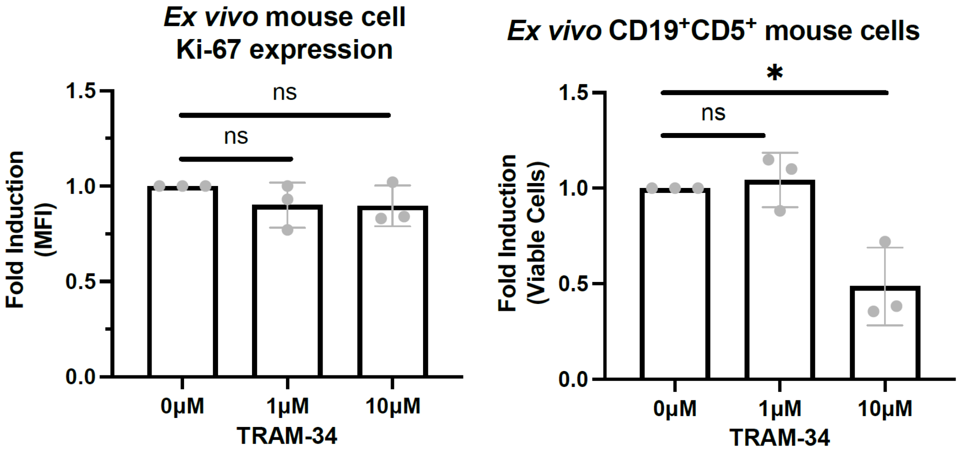

Supplementary Figure 6. Effect of TRAM-34 on proliferation and survival of B-CLL cells from Eμ–*TCL1* mice. Mononuclear cells were isolated from the spleen of three Eμ–*TCL1* mice and incubated in RPMI in the presence of 10% FBS with 1 or 10 μM TRAM-34 for 48h or left untreated. Cells were then assessed for Ki-67 expression by flow cytometry. Cell vitality was assessed by morphology (ssc-fsc).

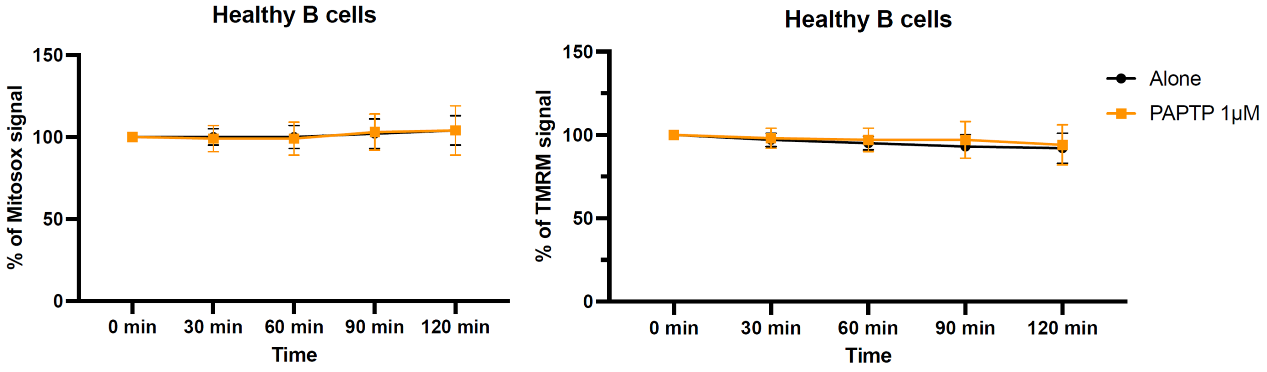

Supplementary Figure 7. PAPTP does not induce ROS release and mitochondrial depolarization in B lymphocytes of healthy subjects. Experiments (n=8 for each panel) were performed as in Figure 6a. "Alone" refers to untreated samples.
